# Exosomes in Cardiovascular Diseases: Pathological Potential of Nano-Messenger

**DOI:** 10.3389/fcvm.2021.767488

**Published:** 2021-11-12

**Authors:** Anshul S. Jadli, Ananya Parasor, Karina P. Gomes, Ruchita Shandilya, Vaibhav B. Patel

**Affiliations:** ^1^Department of Physiology and Pharmacology, Cumming School of Medicine, Calgary, AB, Canada; ^2^Libin Cardiovascular Institute, University of Calgary, Calgary, AB, Canada

**Keywords:** extracellular vesicles, exosomes, cardiovascular disease, heart failure, atherosclerosis

## Abstract

Cardiovascular diseases (CVDs) represent a major global health problem, due to their continued high incidences and mortality. The last few decades have witnessed new advances in clinical research which led to increased survival and recovery in CVD patients. Nevertheless, elusive and multifactorial pathophysiological mechanisms of CVD development perplexed researchers in identifying efficacious therapeutic interventions. Search for novel and effective strategies for diagnosis, prevention, and intervention for CVD has shifted research focus on extracellular vesicles (EVs) in recent years. By transporting molecular cargo from donor to recipient cells, EVs modulate gene expression and influence the phenotype of recipient cells, thus EVs prove to be an imperative component of intercellular signaling. Elucidation of the role of EVs in intercellular communications under physiological conditions implied the enormous potential of EVs in monitoring and treatment of CVD. The EVs secreted from the myriad of cells in the cardiovascular system such as cardiomyocytes, cardiac fibroblasts, cardiac progenitor cells, endothelial cells, inflammatory cells may facilitate the communication in physiological and pathological conditions. Understanding EVs-mediated cellular communication may delineate the mechanism of origin and progression of cardiovascular diseases. The current review summarizes exosome-mediated paracrine signaling leading to cardiovascular disease. The mechanistic role of exosomes in cardiovascular disease will provide novel avenues in designing diagnosis and therapeutic interventions.

## Introduction

Described initially as debris with no functional significance or potential involvement in the clearance of damaged cellular components, extracellular vesicles (EVs) represent a heterogenous population of vesicles. Accumulating evidence suggesting the involvement of EVs in intercellular communication by virtue of the transfer of bioactive molecules invoked scientific interest in the field of EVs (1, 2). Increasing efforts to unravel the mechanisms of biogenesis, molecular composition, physiological and pathological functions of EVs led to the identification of major subtypes of vesicles, their association with pathological conditions, and potential therapeutic applications. Broadly, EVs are classified as exosomes (30–100 nm), microparticles (0.1–1 μm), and apoptotic bodies (1-5 μm) depending on the size, mechanism of biogenesis, and surface markers (3–5). Exosomes are the smallest identified group of EVs implicated in cellular communication. By its attribute to carry and transfer DNA, RNA, miRNA, and protein, exosomes facilitate intercellular communication and thus play a vital role in physiological and pathological conditions (6, 7). Cardiovascular diseases represent a group of debilitating and often fatal diseases affecting the population worldwide. Exosome-mediated signaling has been implicated in the progression of cardiovascular diseases such as heart failure (HF), myocardial ischemia, and atherosclerosis, among others (8–11). The current review will summarize the role of exosome-mediated communication in the development of cardiovascular diseases and its potential as therapeutic targets in cardiovascular pathologies.

## Cardiovascular Diseases (CVDs) and Exosomes

CVDs represent a major health concern resulting in significant mortality and morbidity in developed and developing countries (12–14). Adverse health impact and high economic burden of management prompted to identify novel therapeutic interventions for diagnosis and treatment of CVDs. The complex, multifactorial nature of CVDs and inadequate mechanistic insight of pathogenesis hampered the identification of novel drug targets. Though cardiomyocytes represent one-third of the total number of cells in the heart, different cell types such as fibroblasts, smooth muscle cells (SMCs), endothelial cells (ECs), neuronal cells, inflammatory cells, and cardiac-derived stem cells orchestrate maintenance of physiological functions (8). Recently, the focus has shifted toward identifying the mechanisms of cell-to-cell communication between these cell types in (patho)physiological conditions (15–17). During the pathological conditions, cardiac ECs reported shedding of EVs that facilitate disease progression (18, 19). Similarly, EVs subtypes have been implicated as beneficial markers due to their cardioprotective attributes (20–22). With the elucidation of the function of exosomes as a biological messenger in intracellular communication in the field of hematology, immunology, and oncology, its role as a novel therapeutic target for prognostic/diagnostic biomarker in cardiac pathophysiology has been actively investigated.

### Atherosclerosis

**Figure 1:**
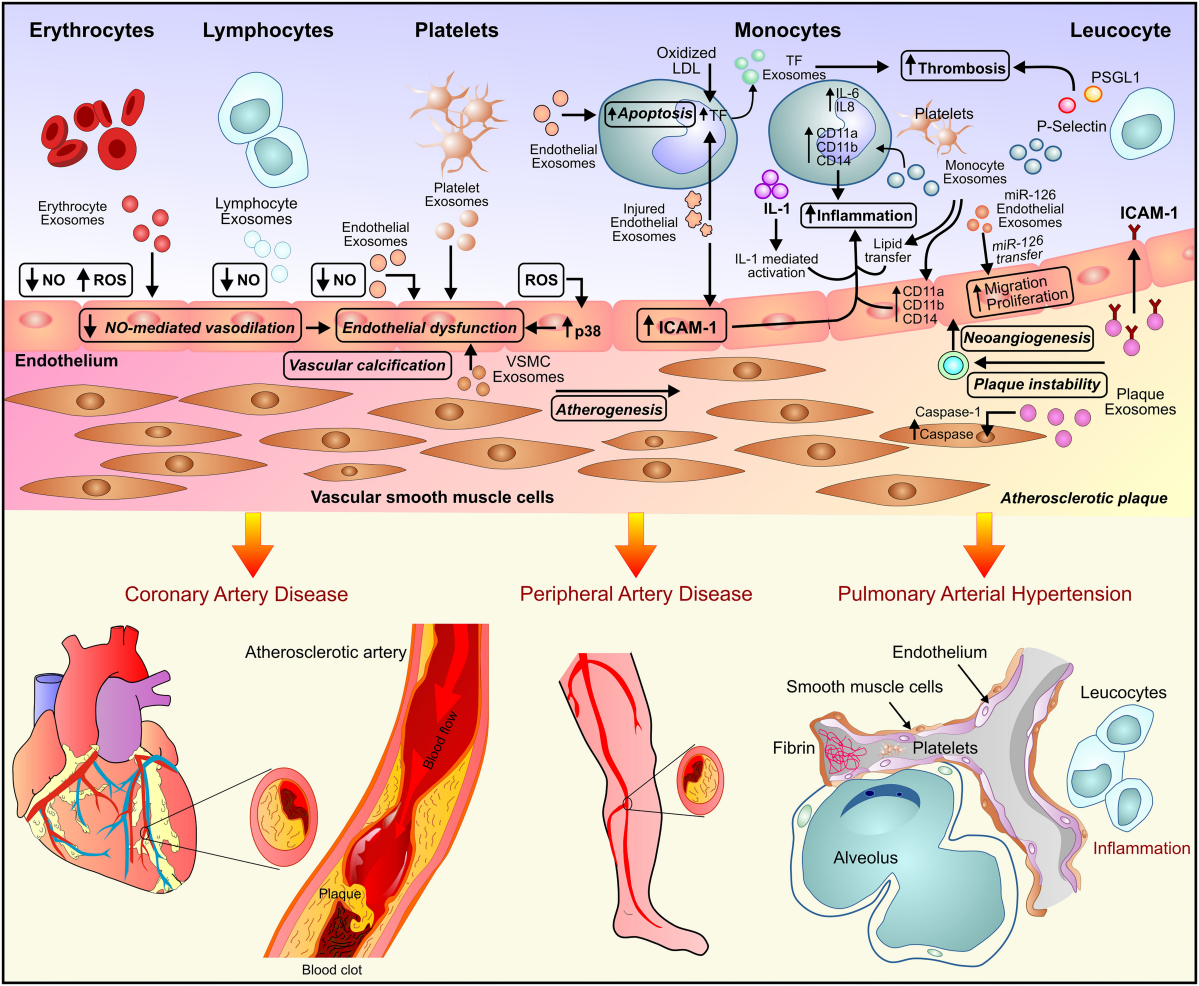
Exosome-mediated paracrine signaling and development of vascular diseases. Exosome secreted by endothelial cells, inflammatory cells, and the myriad of circulating blood cells leads to endothelial dysfunction and inflammation and subsequently, the development of vascular diseases such as atherosclerosis, peripheral artery disease, and pulmonary arterial hypertension.

A subset of coronary artery disease, atherosclerosis is a pro-inflammatory condition characterized by endothelial cell activation, inflammatory cell recruitment, adhesion molecules, vascular smooth muscle cells (VCMCs) phenotype transformation, and plaque formation (Figure 1). In response to the environmental conditions, exosomes released by ECs, VSMCs, immune cells lead to intracellular communication and hence augmentation of the inflammatory response (23, 24). Atherosclerotic plaque-derived EVs reported transferring adhesion molecules such as ICAM-1 to ECs leading to plaque progression *via* the recruitment of immune cells. The EVs-mediated transfer of effector molecules suggested a functional correlation between plaque-derived EVs and plaque development in atherosclerosis (25). Atherosclerosis is associated with chronic inflammation and increased dendritic cells (DCs) which contribute to the activation of ECs through cytokine production. DC-derived exosome reported to be involved in the activation of ECs by TNF-α and NF-kB signaling pathways in human umbilical vein endothelial cells (HUVECs). Activation of TNF-α and NF-kB signaling pathways in HUVECs lead to transcription of adhesion molecules such as VCAM-1, ICAM-1, and E-selectin thus suggesting the involvement of DC-derived exosomes in the activation and inflammation of ECs in atherosclerosis (9).

Elevated levels of Pro-atherogenic inducers, oxidized LDL (ox-LDL), and homocysteine (Hcy) were reported as risk factors for atherosclerosis and cardiovascular morbidities. In rat aortic endothelial cells (RAECs), ox-LDL and Hcy induced the dose-dependent release of HSP70-containing exosomes. The exosome-dependent HSP70 release triggered activation of monocytes, resulting in monocyte adhesion to ECs and hence Pro-atherogenic, pro-inflammatory effects in the onset of atherosclerosis (26). Endothelium dysfunction due to the difference in local blood flow pattern generally results in atherosclerosis initiation. Krüppel-like factor 2 (KLF2) is a key regulator of shear stress-induced atheroprotective endothelial phenotype. Exosome-mediated transfer of atheroprotective miR-143/145 by KLF2-expressing HUVECs to human aortic SMCs (HASMCs) resulted in reduced atherosclerotic lesion formation by repression of miR-143/145 target genes and de-differentiation associated genes (27). Vascular calcification due to the deposition of calcium phosphate salts in the medial and intimal layers of the vessel wall is one of the common complications associated with atherosclerosis. In response to the pathogenic stress conditions, the transition of VSMCs into a procalcified phenotype leads to the pathogenesis of atherosclerosis. The exosomes secreted by proliferative VSMCs were reported to be enriched with fetuin-A. Fetuin-A is a potent calcification inhibitor glycoprotein that binds to minerals and aids in vascular repair processes, like adhesion and migration. The osteogenic stimuli such as elevated cytokines, growth factors, calcium, and mineral imbalance resulted in the upregulation of sphingomyelin phosphodiesterase 3 and promoted the secretion of exosomes. The exosomes thus lead to the process of vascular calcification by shifting proliferative VSMCs toward the procalcified phenotype (28).

Diverse physiological effects were exerted by the platelet-derived exosomes on the inflammation process in response to pathogenic stimuli. Platelet-derived EVs reported being involved in the mitogen-induced proliferation of SMCs in atherosclerosis (29). Similarly, unesterified cholesterol EVs from human monocytes promoted activation of ECs and maladaptive immune response which may contribute to atherothrombosis (30). Monocyte-derived EVs exerted atherogenic effects by activation of ECs and stimulation proinflammatory response resulting in vascular inflammation and atherosclerotic plaque formation (31). The monocyte-derived EVs activate pathways related to nitrosative stress in ECs including PI3-kinase and ERK1/2 in the regulation of caveolin-1 expression. The pleiotropic effects of monocyte-derived EVs on ECs may play an important role in the pathogenesis of atherosclerosis (32).

The platelet-derived exosomes have been reported to exhibit atheroprotective properties *via* prevention of two important factors of atherothrombosis i.e., foam cell formation and activation of platelets in response to damaged blood vessels. The endothelial damage is characterized by the formation of foam cells followed by activation of platelets and recruitment of immune cells i.e., macrophages and T lymphocytes. CD36, a type II scavenger receptor, through microparticle binding and intracellular signaling leads to platelet aggregation and atherothrombosis. Platelet-derived exosomes increase protein ubiquitination and enhance proteasome degradation of CD36. This leads to reduced platelet aggregation and adhesion to collagen matrix which attenuates occlusive thrombosis in a FeCl_3_-induced arterial injury model (33). Expressed by macrophages, CD36 binds and internalizes oxidized LDL thus facilitating foam cell formation (34, 35). Platelet exosomes attenuated foam cell formation by inhibiting CD36 dependent oxidized LDL binding and loading of cholesterol into macrophages (33). Loading of excessive cholesterol in macrophages results in the secretion of the proinflammatory cytokine tumor necrosis factor α (TNF-α), which promotes atherogenesis (36, 37). Activated T lymphocytes release exosomes, enriched in cholesterol and exposing phosphatidylserine (PS) at their outer membrane leaflet. Internalization of cholesterol-enriched exosomes by monocytes induce secretion of TNF-α thus implying a functional association between T- lymphocyte-derived exosomes and atherogenesis (37). Recently, exosome-mediated transfer of miR-155 from VSMCs to ECs has been implicated in the pathogenesis of atherosclerosis. KLF5-overexpressing VSMCs exerted pro-atherosclerotic effect by secretion of miR-155 enriched exosomes. Uptake of these exosomes by ECs resulted in inhibition of cell proliferation and migration and disrupted endothelial barrier integrity. miR-155 mediated downregulation of tight junction proteins such as ZO-1 and claudin 1 lead to an increase in the endothelial permeability and thus the progression of atherosclerotic phenotype. Genetic deletion or inhibition of miR-155 using anti-miR-155 resulted in suppression of atherogenesis, suggesting the pathogenic function of miR-155 in the development of atherosclerosis *via* exosome-mediated transfer (38).

CD137 is a tumor necrosis factor receptor (TNFR) superfamily member which ligates to its ligand, CD137 L for activation of CD137 signaling. Ten-eleven translocation 2 (TET2), is a DNA demethylase, a member of the methylcytosine dioxygenase family that oxidizes 5-methylcytosine (5mc) to 5-hydroxymethylcytosine (5hmc). It plays a crucial role in gene activation through DNA demethylation mediated by the conversion of 5hmc to unmethylated cytosine. TET2 has been reported to be involved in the regulation of SMCs plasticity by inactivating dedifferentiation genes and activating contractile marker genes of SMCs. Activation of CD137 signaling in ECs plays an important role in the induction of immune and inflammatory responses in atherosclerosis. The activation of CD137 inflammatory signaling in endothelial cells (ECs) downregulates TET2 expression in EC-derived exosomes. This CD137 signaling-mediated exosomal downregulation of TET2 expression resulted in a pro-proliferation, phenotype switch of VSMCs, and intimal hyperplasia after arterial injury. The phenotypic switching and intimal hyperplasia of VSMCs induced by activation of CD137 signaling attenuated by overexpression of TET2 expression in EC-derived exosomes. This corroborated that activation of endothelial CD137 signaling promoted neointimal formation by reducing the expression of TET2 in ECs and ECs-derived exosomes, leading to the reduced exosome-mediated TET2 transfer to VSMCs (39). T helpers 17 (Th17), a subset of lymphocytes, along with the associated cytokinesis have been implicated in the pathogenesis of atherosclerosis. The effects of CD137 signaling in inflammation and atherosclerosis progression *via* regulation of Th17 cell responses have been explored *in-vitro* and *in-vivo*. Exosomes derived from CD137 modified ECs (CD137-Exo) increased the proportion of Th17 cells *in-vitro* and *in-vivo*. Regulated by Akt and NF-kB pathways, IL-6 contained in CD137-Exo activated Th17 cell differentiation. Lipopolysaccharide (LPS)-induced inflammation in ECs showed IL-17-mediated apoptosis, inhibited cell viability, and increased lactose dehydrogenase (LDH) release. CD137-Exo promoted Th17 cell differentiation *via* the NF-kB pathway-mediated IL-6 expression and atherosclerosis progression (40).

Exosomes derived from high fat diet-visceral adipose tissue (HFD-VAT) *via* downregulation of ATP-binding cassette transporter (ABCA1 and ABCG1)–mediated cholesterol efflux facilitated macrophage foam cell generation. HFD-VAT exosome-induced increased lipid accumulation in macrophages was associated with decreased cellular levels of LXR-α, a nuclear receptor known to mediate the transcription of ABCA1 and ABCG1. Thus, exosomes exerted the dual effects of repression of ABC transporter expression as well as cholesterol efflux. *In vitro*, HFD-VAT exosomes induced M1 phenotype transition and proinflammatory cytokine (TNFα and IL-6) secretion in macrophages. Intravenous administration of HFD-VAT exosomes to hyperlipidemic apolipoprotein E–deficient mice leads to exacerbated atherosclerosis, suggesting their proatherogenic effects by modulation of foam cell formation and M1 phenotype polarization of macrophages (41). In the study investigating delivery free fatty acids (FFAs) from the bloodstream to cardiac tissue, the results demonstrated the exosome-mediated mechanism of circulating FFAs delivery from blood to tissue. Circulating exosomes from healthy donors express fatty acid transporter, CD36 which by binding to long-chain FFAs facilitates its transport into the cells. Increased exosomal CD36 levels have been reported in exosomes harvested in postprandial (PP) state as compared with the fasting (F) resulting in increased uptake and subsequent delivery of FFAs into cardiac cells (42). Though previously CD36 has been implicated in metabolic dysregulation-associated pathological conditions (43–45), further studies are recommended to elucidate the role of CD36-mediated exosomal delivery of FFAs in the pathogenesis of obesity, diabetes, and atherosclerosis.

Exosomes derived from macrophage THP-1 cells upon treatment with ox-LDL were enriched in miR-146a. Exosomal miR-146a promoted reactive oxygen species (ROS) generation and Neutrophil extracellular traps (NETs) released *via* downregulation of superoxide dismutase 2 (SOD2) expression. Intravenous administration of miR-146a enriched exosomes derived from oxLDL-treated THP-1 cells to a murine model of atherosclerosis exhibited exacerbation of atherosclerosis. This suggested exosomal miR-146a-induced promotion of atherosclerosis *via* cellular oxidative stress and NETs formation (46). By inducing oxidative stress and endothelial dysfunction, ox-LDL contribute to the development of atherosclerosis (47, 48). *In-vitro* activation of the NF-κB pathway was observed in HUVECs treated with ox-LDL, which transcriptionally activates miR-505. Significant upregulation of miR-505 expression in HUVECs and HUVECs-derived exosomes inhibited SIRT3 in neutrophils by targeting the SIRT3 3'UTR. Inhibition of SIRT3 increases the reactive oxygen species (ROS) levels, and subsequently the release of NET by neutrophils. *In vivo* studies involving the atherosclerotic murine model showed accelerated progression of atherosclerosis upon administration of ox-LDL-exosome (49). The interactions between ECs and macrophages play a crucial role in cardiovascular homeostasis and the development of atherosclerosis. Exosomes derived from ox-LDL-treated ECs were found to be enriched in miR-155, which is known to be in association with inflammatory responses in macrophages. Exosome-mediated transfer of miR-155 to monocytic cell line THPI leads to the activation of monocytes by polarization toward proinflammatory M1 macrophages. Thus, the study provided evidence suggesting exosomal miR-155-mediated regulation of inflammation associated with atherosclerosis progression (50).

The study investigating the role of hepatocyte-derived EVs in distant communication between liver and vasculature showed hepatocyte-derived exosome-mediated promotion of atherosclerosis. Hepatocyte-derived exosomes exacerbated endothelial inflammation and facilitated atherogenesis by miR-1 delivery. The atherogenic effects of miR-1 were attributed due to the downregulation of KLF4 and NF-kB pathway activation in ECs. Inhibition of miR-1 attenuated endothelial inflammation and atherogenesis suggesting the role of hepatocyte-derived exosomes in the pathogenesis of vascular complication (51). A recently mechanistic insight of the association between *H. pylori* PMSS1 infection and the progression of atherosclerosis revealed exosomal CagA-mediated pathogenic progression. *H. pylori* PMSS1 is a CagA-positive strain that can translocate CagA into host cells. Through Type-IV secretion system (T4SS) translocation into host cells, CagA is a major virulence factor that is involved in upper gastrointestinal diseases. CagA-positive *H. pylori* infection did not initiate but aggravated the progression of atherosclerosis *via* exosome CagA-mediated macrophage foam cell formation. *H. pylori*-infected gastric epithelial cells-derived exosomes (Hp-GES-Exo) promoted macrophage-derived foam cell formation and lesion development *via* exosomal CagA *in-vitro* and *in vivo*. Mechanistically, exosomal CagA downregulated the expression of transcriptional factors PPARγ and LXRα and led to the suppression of transcription of cholesterol efflux transporters. Thus, the study elucidated another mechanism i.e., the effect of *H. pylori* infection on the progression of atherosclerosis (52). Nicotine has been implicated in the induction of atherogenesis *via* modulation of the proatherogenic phenotype of VSMCs. Exosomes from nicotine-stimulated macrophages (NM-Exo) promoted VSMCs migration and proliferation. NM-Exo was found to be enriched in miR-21-3p which modulated the proatherogenic phenotype of VSMCs by regulation of phosphatase and tension homolog (PTEN). The study suggested nicotine-stimulated macrophages facilitated atherosclerosis progression by enhanced VSMCs migration and proliferation *via* exosomal miR-21-3p- mediated regulation of PTEN (53).

The maturation of dendritic cells (DCs) has been implicated in the development of atherosclerosis. Involved in tumorigenesis, Metastasis- associated lung adenocarcinoma transcript-1 (MALAT1) is a long non-coding RNA (lncRNA), and its downregulation promotes atherosclerosis. *In-vitro* and *in-vivo* experiments explored the effect of exosomal MALAT1 in the maturation of DCs and subsequently in the progression of atherosclerosis. Exosomes derived from HUVECs treated with ox-LDL exhibited reduced MALAT1 expression. Exogenous overexpression of MALAT1 in exosomes- derived from ox-LDL treated HUVECs transferred elevated MALAT1 levels to immature DCs. This exosomal MALAT1 interacted with the nuclear factor erythroid 2-related factor (NRF2) and activated NRF2 signaling, which inhibited ROS accumulation and subsequently DCs maturation. *In vivo* experiments suggested an association between atherosclerosis progression and decreased MALAT1 content in VECs-exosomes. Thus, loss of MALAT1 expression in ox-LDL-treated VECs- derived exosomes lead to the maturation of DCs in the development of atherosclerosis (54). Exosomes derived from HUVECs treated with ox-LDL showed upregulation of MALAT1 expression. Human neutrophils upon treatment with exosomes from ox-LDL-treated HUVECs exhibited NETs formation *via* activation of the P38/Akt signaling pathway induced by exosomal MALAT1. Administration of exosomes derived from ox-LDL-treated HUVECs to a murine model of atherosclerosis triggered hyperlipidemia, inflammation, and NETs release indicating exacerbation of atherosclerosis (55).

Numerous studies reported the modulatory function of lncRNAs in the regulation of proliferation, migration, inflammation, apoptosis, and calcification (56). High expression of lncRNA GAS5 had been implicated in atherogenesis by regulation of apoptosis of macrophages and ECs (57). Similarly, MALAT1 affected inflammation of ECs through SAA3 and was identified as a regulator of inflammatory cytokine production (58). Exosomal lncRNA-p21 was identified as a novel regulator of neointimal formation, cell proliferation, and apoptosis in atherosclerosis. Subsequently, a recent study reported that inhibition of lncRNA-p21 leads to reduced apoptosis of VSMCs by altering p53 activity (59). An *in-vitro* study reported the role of exosome-mediated lncRNA ZEB1-AS1 and its underlying mechanisms in atherogenesis in HUVECs. In ox-LDL-stimulated HUVECs, exosomal lncRNA ZEB1-AS1 facilitates cell injury by miR-590-5p/ETS1 Axis through the TGF-β/Smad Pathway (60). As phenotypic switch of VSMCs predisposes development of atherosclerosis, Zhang et.al. showed that exosomal LINC01005 derived from ox-LDL-treated HUVECs promotes VSMCs phenotypic switch, proliferation, and migration *via* regulation of the miR-128-3p/KLF4 axis (61).

Biosynthesis of Leukotrienes (LTs), potent proinflammatory lipid mediators, can be induced by exosomes (62–64). Essar et al. demonstrated that exosomes from macrophages and dendritic cells (DCs) contain functional enzymes for LT biosynthesis. Thereby, exosomes can contribute to inflammation by participation in LT biosynthesis and granulocyte recruitment (62). DCs primarily formed LTC4 while macrophages produced LTA4 and LTB4. LTB4 is derived from the 5-lipoxygenase pathway of arachidonic acid metabolism and has a proinflammatory effect by activation of G-protein coupled receptors (65, 66). Exosomes derived from neutrophils contain LTB4 and LTB4-induced enzymes. Being a chemoattractant, LTB4 can stimulate the accumulation of macrophages at the inflammation site (67). Neutrophils release chemotaxis to endothelial cells during atherosclerosis. The neutrophil-mediated process can aggravate endothelial dysfunction and cause macrophages to accumulate in the vulnerable vessel walls (68). Although these studies provide key evidences, understanding the role of exosomal lipid transport in the onset and progression of CVD is extremely limited and warrants further investigations.

### Heart Failure

HF is a progressive condition marked by the inefficiency of the heart to pump enough blood to support the physiological demand of the body. It is characterized by signs and symptoms involving multiple organ systems such as the heart, liver, kidney, and lungs. The abnormal cardiac remodeling in HF includes cardiomyocyte hypertrophy, fibrosis, aberrant angiogenesis, and infiltration of inflammatory cells (69). Based on ejection fraction (EF), HF can be classified as HF with reduced EF (HFrEF) or HF with preserved EF (HFpEF). Coronary artery disease with underlying systemic inflammation, atherosclerosis, and comorbidities represents major risk factors in patients with HFrEF (70). Increased plasma levels of pro-inflammatory cytokines in HFrEF patients, and Toll-like receptor-dependent determination of post-MI EF decrease in the murine model suggested a role of systemic inflammation in LV remodeling post-MI (71, 72). HFpEF is a pro-inflammatory state associated with comorbidities such as obesity, salt-sensitive hypertension, diabetes mellitus, and chronic obstructive pulmonary disease. The systemic inflammation induced by these comorbidities leads to adverse structural and functional alteration in the myocardium. The systemic inflammatory state results in coronary microvascular endothelial inflammation, hypertrophy, increased diastolic left ventricular (LV) stiffness, and thus, HF (73).

As a systemic vascular disease with the underlying pro-inflammatory state has been implicated in development in HF, in an observational study comprising dyspnoea patients, EV markers have been investigated in patients with or without HF. The study reported the association of extracellular vesicle levels of systemic vascular markers such as CD14, SerpinG1, and SerpinF2 with the development of HF in patients suspected of acute HF (74). A recent study demonstrated increased exosomal levels of inflammation-associated miRNAs i.e., miR-146a and miR-486 in HF patients. In response to inflammation in HF patients, miR-146a and miR-486 levels were increased for attenuation of inflammatory targets and thus imparting cardioprotective effects against oxidative stress in HF (75). Disruption in cellular redox signaling and macromolecular damage has been attributed to an imbalance in ROS generation and subsequent adverse cardiac structural and functional alteration. This can lead to myocardial hypertrophy, fibrosis, and contractile dysfunction in chronic HF. The Keap1-Nrf2 pathway is a major regulator of antioxidant protective mechanisms against ROS and electrophiles mediated endogenous and exogenous stress. Recently, Nrf2 dysregulation mediated by miRNA enriched exosomes has been reported in the pathogenesis of CHF. Downregulation of 6 weeks post-MI protein levels of Nrf2 has been observed in the heart with increased Nrf2 transcription. This suggested miRNA-mediated suppression of Nrf2 translation. Exosome-associated microRNA-27a,−28-3p, and−34a were expressed in the left ventricle of the infarcted heart. The miRNAs enriched exosome-mediated intercellular communication in Nrf2 dysregulation in response to MI subsequently results in oxidative stress and development of CHF (11).

Cardiac Hypertrophy represents an important subtype of adaptive cardiac remodeling in response to cardiac overload. The condition is characterized by cardiomyocyte enlargement, secretion of inflammatory factors, fibroblast proliferation, and extracellular matrix protein secretion. The hypertrophic cellular responses are due to extracellular vesicle-mediated cell-cell communication between cardiomyocytes and other cells of the myocardium such as ECs, fibroblasts, and inflammatory cells. Under stressed conditions, the upregulation of exosome release with enriched pro-inflammatory and apoptotic markers by adult cardiomyocytes leads to inflammation and cardiomyocyte death which further exacerbates inflammatory response (76). An *in-vitro* study investigating the effect of TGF- β on the development of HF suggested the onset of HF phenotype in cardiomyocytes *via* TGF- β-treated cardiac fibroblast-derived exosomes. Cardiomyocytes co-cultured with exosomes from TGF-β treated fibroblasts and fibroblasts from HF patients exhibited differentially expressed genes and transcriptional changes associated with hypertrophic cardiac cells. In concordance with the previous animal studies reporting fibroblasts exosome-induced cardiac hypertrophy (77), pathway analysis showed activation of miR-155 and miR-21 in exosomes from TGF-β treated fibroblasts and regulation of signaling associated with cardiac cardiomyocytes (78).

Chronic activation of the myocardial renin-angiotensin system (RAS) and subsequent increased angiotensin II (Ang II) levels have been implicated in pathological cardiac hypertrophy. Treatment of cultured cardiomyocytes fibroblasts (CFs) with Ang II enhanced the release of exosomes by activation of Ang II receptor type 1 (AT_1_R) and 2 (AT_2_R). The CFs derived exosomes upregulated the expression of renin, angiotensinogen, AT_1_R, and AT_2_R while downregulated expression of angiotensin-converting enzyme 2 and increased production of Ang II in cultured cardiomyocytes thus overall exacerbated Ang II-induced cardiac hypertrophy. Ang II-induced release of CFs derived exosomes, therefore, suggested a paracrine mechanism of Ang II-mediated cardiac hypertrophy (79). Adipose tissue has been implicated in systemic homeostasis and cardiac hypertrophy *via* interaction with cardiomyocytes. Activation of PPAR-γ in adipocytes leads to the expression and exosomal release of miRNA-200a. The exosomal miRNA-200a target cardiomyocytes and induce hypertrophy by suppression of TSC1 and mammalian target of rapamycin (mTOR) pathway activation (80). The exosome-mediated communication between cardiomyocytes and cardiac fibroblasts has been investigated in the pathogenesis of cardiac hypertrophy. In an *in-vitro* study, Cardiomyocytes (CMs) co-cultured with fibroblasts or fibroblast conditioned media (FCM) demonstrated enhanced hypertrophy. The cardiomyocyte hypertrophy and CM regulated fibroblast adhesion/ proliferation was attributed to the paracrine interaction between CMs and fibroblasts (81). Enriched with miR-21-3p (miR-21*), cardiac fibroblast-derived exosomes promoted cardiac hypertrophy upon temperature and actin-dependent uptake by cardiomyocytes. In *in-vivo* Ang II-induced cardiac hypertrophy mouse model, pharmacological inhibition of miR-21* attenuated hypertrophy (77). Further, miR-21* mediated downregulation of sorbin, SH3 domain-containing protein 2 (SORBS2), and PDZ and Lim domain 5 (PDLIM5) in cardiomyocytes proposed as a mechanism of development of cardiomyocyte hypertrophy. Significantly elevated levels of miR-21* were observed in the pericardial fluid of mice with left ventricular pressure overload-induced hypertrophy after the aortic constriction. The study suggested miR-21* as a paracrine mediator of cardiomyocyte hypertrophy and a potential therapeutic target in HF (77). Other studies reported the exosome-mediated transfer of miRs such as miR-214, miR-21, miR-145-5p, miR-135b, and miR-125a in angiogenesis by up/downregulation of target gene expression in ECs (82–87).

In response to a hypoxic condition, CPCs derived exosomes showed angiogenic and antifibrotic potential and lead to tube formation of ECs and decreased profibrotic gene expression in TGF-β-stimulated fibroblasts. *In vivo* administration of CPCs derived exosomes leads to improvement of cardiac function and reduced fibrosis in the Ischemia-reperfusion rat model (88). Association of cardiosphere derived exosomes with cardiac regeneration and cardiac protection by rendering angiogenic, antifibrotic, and anti-apoptotic properties to recipient cells have been reported previously (22, 89). Treatment of HFpEF rat model with cardiosphere derived cells (CDCs) resolved diastolic dysfunction evidenced by decreased inflammation and LV fibrosis. Though CDCs treatment did not attenuate hypertrophy and hypertension; improved survival due to normalized LV function suggested CDCs as a potential treatment for HFpEF (90). Thus, these reports suggested CPCs derived exosomes as potential therapeutic agents against heart damage.

### Ischemic Heart Disease

Acute MI is defined by a block in blood flow, usually in the coronary arteries, which causes immediate cardiac damage, cell death due to ischemia, and subsequently, cardiac remodeling that negatively impacts heart function. Current treatments such as coronary bypass surgery or balloon dilatation of coronary vessels focus on the initial cardiac remodeling and acute stage of MI, rendering them ineffective on subsequent cardiac damage (91–94). Identifying novel targets that increase tissue repair and regeneration, preserve vasculature, and increase immune clearance can provide a therapeutic avenue (94). After acute myocardial infarction (MI), the myocardium accumulates a larger mass and has to work harder to pump blood through the heart. The heart undergoes adaptive actions due to signals from chemical signals/factors such as chemokines, cytokines, growth factors, proteins, mRNAs, and miRNAs, all of which are enclosed in exosomes derived from cardiac cells (95). Exosomal small regulatory non-coding miRNA (micro RNA proteins) have been seen to be altered after MI (96, 97). The exosomal cargo consists of signaling molecules that can impact micro communication with local or distant tissues/organs (97). This miRNA has been previously termed “exosomal shuttle RNA” or esRNA (98). Stem and progenitor cells release exosomes enriched with miRNAs to maintain cardiac remodeling and regeneration post-MI (93). CPC-derived exosomes have therapeutic benefits, promoting angiogenesis *via* an EMMPRIN-mediated mechanism (extracellular matrix metalloproteinase inducer) (99). Hypoxic CPC-derived exosomes especially have angiogenic potential, increasing tube formation of ECs, myocardial mass, and decreasing profibrotic gene expression, infarct size, pathological cardiac remodeling, and apoptosis (96, 99). The miRNAs such as miRNA-214, 133a, 21, 34a, 208, 1, and 92a can impact local myocardial cells i.e., cardiomyocytes, ECs, fibroblasts, and stromal cells. The exosome-associated miRNAs such as miRNA-126, 130a, 150, and 34a can reprogram the BM after an ischemic injury to release more BM cells (97) and show significantly reduced levels in patients with HF (95). Increased cardiac-derived exosomal secretion has been reported in cases of AMI associated with hypoxic stress (100).

The identity and concentrations of exosomal cargo vary in MI (101). miRNA 126, 125a, 17, 19a, 19b, 30c, 31, 150, 296, and 214 are known to stimulate angiogenesis (92, 99, 102). Angiogenic miRNAs such as miR-30c, 126, 17, and 19a/b derived from cardiomyocytes, undergoing glucose-restricted states for 48 hours, caused tube formation *via* the increased proliferation of HUVECs (101). Low circulatory levels of miR-126 in acute MI patients indicated vascular damage and associated reparation process due to the uptake of proangiogenic endothelial cell-derived miR-216 by the damaged myocardium (95, 97, 102, 103). This suggested miR-126 as an MI signature in patients, and activation of miR-126 implicated in increased vascular injury and cardiovascular risk (95, 97) as it regulates vessel integrity and angiogenesis (96). miR-126 has been reported in regulation of the expression of CXCL12 (C-X-C motif chemokine 12, the receptor for stromal cell-derived factor 1/SDF1) through a feedback loop (101), which further promotes the recruitment and mobilization of BM-derived stem cells into the heart (97). The function of human circulating peripheral blood-derived CD34^+^ staminal exosomes is enriched with the upregulation of miR-126, which results in increased exosomal release from stem cells (97). In turn, these CD34^+^ exosomes usually contain proangiogenic hematopoietic stem cell-derived miR-126 and miR-130a, contributing to the angiogenic capabilities of the exosomes (101).

The exosomal proangiogenic miR-126-3p reported being involved in atheroprotection by increased *in-vitro* and *in-vivo* progenitor cell mobilization by inhibiting RSG16 (regulator of G protein signaling), thus upregulating CXCR4 and CXCL12 signaling (18, 102, 104). Stable miR-126-3p is transferred between cells, inhibiting expression of its direct target SPRED1 (Sprouty-related, EVH1 domain-containing protein 1), thereby controlling genes regulating angiogenesis such as VEGF (vascular endothelial growth factor), MMP9 (matrix metallopeptidase 9), ANG1 (angiopoietin 1), ANG2 (angiopoietin 2), and TSP1 (thrombospondin 1) (104). An increase in miRNA-126-3p and erythropoietin-dependent pSTAT5 in the recipient ECs *via* exosomal delivery inhibits SPRED1 and upregulates ERK 1/2 and cyclin D1 transcription (102), to cause more EC proliferation and migration for re-endothelialization after ischemic damage (18). In the ischemic conditions after AMI, pro-angiogenic and anti-apoptotic genes are downregulated, but this is restored *via* uptake of exosomal angiogenic miRNAs by ischemic ECs, a process controlled by L-selectin (102). Another proangiogenic miRNA, miR-125a, when transferred to cardiac cells *via* exosomes works to regulate the number and differentiation specifications of stem cells. miR-125a directly suppresses the Notch ligand δ-like 4, downstream of VEGF, causing the formation of capillary-like structures *in-vitro* and *in-vivo*. On the other hand, miR31 delivery inhibits the factor suppressing HIF-1a (hypoxia-inducible factor 1), improving the environmental conditions in the damaged myocardium (101, 102). Inhibition of miR-126 and miR-296 resulted in attenuation of the renoprotective effect evident by reduced capillary density, more cardiac tissue destruction, and no angiogenic stimulation (102). The hypoxic conditions in AMI stimulate enrichment of exosomes with miR-126 and miR-210, which upon internalization by hypoxic ECs initiate PI3K/Akt, Akt/GSK3, and other pro-survival pathways, reducing inflammation and c-JNK signaling, thus increases the resistance of stem and progenitor cells (like CPCs) to hypoxic stress (96, 101). miRNA-210 downregulates caspase-8-associated protein 2 and ephrin A3, having anti-apoptotic impacts. Another pro-survival miRNA, miR-120, is instigated by hypoxia (96). miR-120 internalization decreases mitochondrial metabolism (101).

During AMI, exosomal cargo is altered and engineered to contain more cardioprotective factors such as miRNA (214, 1, 208, 22, 133a) or heat shock protein 70 (Hsp70) from BM derived-stem cells (93, 95). In non-MI healthy controls, the cardioprotection is mediated by exosome-mediated delivery of Hsp70 and other endogenous protective signals to the myocardium (93). miR-214 has been upregulated after ischemic tissue damage, being released from EC-derived exosomes, and serves as a “biomarker” for the detection and severity of coronary artery disease (69, 95, 97, 99). miR-214 inhibits the mRNA encoding Na^+^/Ca^2+^ exchanger 1, which controls Ca^2+^ influx and downstream signaling that can play a role in cell death. By increasing miRNA-214 levels after MI, which regulates calcium overload, existing cardiac cells are protected, and the contractility and cardiac function of the heart is maintained (97, 99). miR-214 also increases *in-vitro* and *in-vivo* angiogenesis in human and murine ECs, by suppression of ATM (ataxia-telangiectasia mutated), which regulates cell cycle checkpoints, thus protecting against senescence (95, 102, 105).

After AMI, higher levels of miR-1 and miR-208 were detected in the urine of MI patients and peripheral blood of rats (95). However, even though urine is a non-invasive measure of miRNA levels, different rates of kidney metabolism and urine formation limit the accuracy of measurement (101). Cardiac-specific miRNA such as miR-1, secreted by damaged cardiomyocytes, exhibits antioxidant properties and facilitates cell proliferation, cardiac-specific stem cell differentiation, and organogenesis while reducing oxidative stress and hypertrophy (96), providing an AMI biomarker with high sensitivity and specificity (99). miR-1 mediates post-transcription regulation of Hsp60 and Hsp70 proteins (69) and suppresses CXCR4 expression in BM cells, to mobilize them to the bloodstream toward the site of injury, for protection of existing, surviving myocardium *via* remote ischemic preconditioning (RIPC) mechanism (99). Under hypoxic conditions, cardiomyocytes secrete exosomes enriched with Hsp60 which binds to Toll-like receptors and mediates cardiomyocyte apoptosis *via* TLR4-MyD88-p38/NF-κB pathway. Similar to acute ischemic injury, exosomes enriched with Hsp20, and TNF-α (tumor necrosis factor) were secreted by cardiomyocytes cultured in hypoxic conditions (95). Hsp70 has been implicated in cardioprotection as it interacts with TLR4 to activate a downstream pathway that involves protein kinases 1 and 2 and p38 MAPK, causing Hsp27 phosphorylation, which protects against IR injury (99). Hypoxic, compared to normoxic, exosomes increased proliferation and migration in ECs, causing more tube formation, less fibrosis, and better heart function (96).

In ischemic conditions, mesenchymal stem cells (MSCs) derived miR-22 enriched exosomes resulted in reduced apoptosis of cardiomyocytes. Delivery of miR-22 to fibroblasts under hypoxic conditions leads to reduced cardiomyocyte apoptosis and fibrosis with increased angiogenicity of cardiomyocytes post-MI (93, 95). The anti-apoptotic and reparative properties of miR-22 are due to the downregulation of pro-fibrotic Mecp2 (methyl CpG binding protein 2) (95, 101). The downregulation of pro-fibrotic Mecp2 is also mediated by human perivascular pericytes (“Bristol pericytes”) derived exosomal miR-132 upon transfer to ECs. The protective effect of exosomal miR-132 was evident in a murine model of MI as Bristol pericytes preserved cardiac function *via* decreased fibrosis and promoted angiogenesis and blood flow (101), especially when TGF-β is available, which initiates miR-132. miR-132 increases angiogenesis by downregulating p120RasGAP and upregulating the previously repressed pro-angiogenic Ras pathway (96). Another cardiac fibroblast-derived exosomal protein (such as miRNA-132) is miRNA-21_3p (miRNA-21*), a “star” pro-fibrotic and pro-hypertrophic miRNA that induces cardiac hypertrophy *via* its targets of SORBS2 (sarcoplasmic protein sorbin and SH3 domain-containing protein2) and PDLIM5 (PDZ and LIM domain 5) (95, 96, 101). These 2 targets normally regulate myocardial structure and function, with their inhibition causing hypertrophy (96). Exosomal miRNA-21* regulates cell hypertrophy *in-vitro* and is upregulated 8x after just 14 days of cardiomyocyte hypertrophy (69, 92, 95). Fibroblast-derived exosomes enriched with miR-21-3p* also target cardiac muscle cells and suppresses sorbin and SORBS2 (SH3 domain-containing protein 2), and PDZ and PDLIM5 (LIM domain 5), causing hypertrophy (77, 99). They are endocytosed by cardiomyocytes rather than simply interacting with the cell membrane of cardiomyocytes (92). miR-21 (along with miR-26a) also regulates cell survival *via* controlling MMP-2 expression, thus controlling ECM remodeling during cardiac hypertrophy (69, 95). MMPs can suppress the endogenous G-protein signaling inhibitor (RGS16) (regulator of G-protein signaling 16) (106, 107) to start an autoregulatory feedback mechanism that increases CXCR4 and CXCL12. This results in increased recruitment, homing, and mobilization of progenitor cells, especially in the infarct border zone, to increase cardiac function and repair after MI (107). CXCR4 (chemokine C-X-C motif receptor 4) is the receptor for SDF1 (stromal cell-derived factor) on BM cells (108). SDF1 is upregulated in ischemic conditions, resulting in increased CXCR4 to recruit and home vascular progenitor cells to the damaged areas, to repair and reendothelialize them (101, 108, 109). MMPs are initiated extracellularly by exosomal CD147 to have an impact on EC migration (96).

Exosomes can also have anti-inflammatory impacts by transferring miRNA-222 into recipient cardiac cells to lower endothelial ICAM-1 expression (18). In the transcoronary circulation, anti-apoptotic miR-499 and miR-133a levels are elevated after being released from the damaged myocardium (95, 96, 99). Muscle-specific miR-133a and miR-1 are also elevated in serum during AMI, along with cardiac blood protein biomarker troponin T (97), after being released from telocytes in the border zones and infarct areas (92, 97). miR-133a and miR-1 levels can be determined early on during AMI without there needing to be a subsequent increase in troponin T or creatinine phosphokinase (99, 110). miR-133 and 1 mostly concentrate in exosomes from the ischemic myocardium and can be taken up by other cells, including adjacent surviving cardiomyocytes to shield against hypertrophy (99, 110). Overall, miR-133a supports cell growth, cardiac differentiation, and survival while reducing fibrosis, hypertrophy, and apoptosis (69, 96). miR-133 regulates the IP3 receptor, allowing for calcium signaling that increases hypertrophy (95). miR-133a can be derived from and can also impact cardiac H9C2 cells, part of an embryonic cardiomyocyte cell line (69, 97). miR-1 also inhibits CXCR4 expression in BM mononuclear cells, increasing infarct size (99). In a murine MI model, anti-apoptotic miR-133a levels were low in infarct and peri-infarct areas, with more cardiomyocyte death (69, 92). Anti-fibrotic miRNA-133a can be taken in by non-infarcted cells to inhibit cardiomyocyte hypertrophy (69, 96). Thus, exosomes enriched with miR-133a act as biomarkers for myocardial damage or cardiomyocyte death (in AMI), as they can be present in the urine, unlike troponin T (69, 93, 96).

In MI, miR-133a/b, 1, and 499-5p are upregulated while miR-122 and 375 are downregulated (96, 101). miR-133a/b is especially expressed in late-stage hypertrophy in the muscle (95). Cardiac troponin T and serum creatine phosphokinase levels are elevated simultaneously along with levels of miR-499 and 208b in the plasma, indicative of cardiac injury (101). miR-208 a/b can contribute to cell growth and sarcomeric gene expression (96) whereas miR-499 inhibits apoptosis *via* myosin heavy chain regulation during hypertrophy (69, 95). All these miRNAs are implicated in reducing cardiomyocyte death, cardiac fibrosis, and infarct size while increasing angiogenesis, cardiomyocyte proliferation, and cardiac contractility. The damaged myocardium releases exosomes that modify and reprogram the microenvironment of the BM *via* crosstalk. There is a change in the phenotype to release more BM-derived cells in the systemic circulation. For example, acute ischemia causes the miR-150 and thus its target c-MYB (106) to decrease (95, 101, 109, 111), activating CXCR4 in BM mononuclear cells (BM-MCs), causing their mobilization (18, 103, 112). These BM-MCs stimulate the release of myocardial exosomes which reprogram the BM microenvironment (change cellular phenotype) (103) to begin repair after AMI (92, 113). miRNA-34a, miRNA-192, and miRNA-194 were all seen to be elevated in HF within a year after MI (69, 92, 99, 114).

Depending on the pathological system at play and the cell of origin of the exosome (s), the miRNA inside them can behave differently. For example, in peripartum cardiomyopathy, prolactin-induced miRNA-146a results in increased apoptosis and decreased proliferation (92, 96). Exosomes concentrated with miRNA-146 released from damaged cells are taken up by the cardiomyocytes which activate pathways overseen by ERBB4 (Erb-b2 receptor tyrosine kinase 4), IRAK1 (interleukin-1 receptor-associated kinase 1), and Notch1. This overexpression causes contractility and metabolic defects in the heart, with vessel density decreasing, leading to cardiac hypertrophy (96). However, in a murine model of MI, cardiosphere-derived cell exosomes enriched with miRNA-146a cause increased compensatory angiogenesis, cardiac regeneration, and cardiac function (92, 101). miR-146a inhibits CARD10 (caspase recruitment domain-containing protein 10), an inhibitor of NF-κβ and angiogenesis (96).

miRNA-29 family members such as miRNA-29b and miRNA-455 can downregulate the expression of MMP-9, resulting in less ECM degradation, and more fibrosis and cardiomyocyte uncoupling, which prevents mitochondrial-induced cardiomyocyte death (92, 101). MMP-9, a component of ECM destruction, releases endogenous angiogenesis inhibitors like αVβ integrin (92, 115). MMPs go into transformed cells to destroy scar tissue. MMPs are controlled by miRNAs (69). In ischemic conditions, exosomal miRNA29a are released from the border zones of infarct areas, and their levels in serum are elevated. Also, miRNA-29b and miRNA-455 levels are elevated in regions besides cardiomyocytes, due to less release of MMP9, causing more fibrosis and cardiomyocyte uncoupling (92). Thus, the activation of ECs by exosomes may be due to the exosomal miRNA inhibiting MMP-9 (115). Hypoxia can also induce C2C12 (skeletal myoblast) expression, upregulating the release of miRNA-29a-rich exosomes (92, 101). Skeletal myocytes can form cardiomyocytes and repair the myocardial hypoxic tissue (107). Exosomes from these skeletal myocytes when released into the blood and reaching ischemic cardiomyocytes in the heart or other organs, the miRNAs within them can manage autophagy, a defensive mechanism against apoptosis (92). miRNA-29 controls p53 which initiates apoptosis (69). miR-29a and IGF-1R (insulin-like growth factor 1 receptor) act as protective factors from fibrosis, preventing cardiac remodeling (99) *via* repeated RIC. miRNA-29a inhibits ADAM12 and ADAM19, which are disintegrin proteases. miRNA-29a also inhibits the pro-fibrotic TGF-β-dependent signaling pathway, by inhibiting TGF-β and TAB1 (TGF-β-activated kinase 1-binding protein 1) (96). High levels of miR-29 and IGF-1R found in non-infarcted areas of the heart indicate their presence there contributing to less LV remodeling, oxidative stress, and overall cardiac dysfunction. In AMI, miRNA-30a controls autophagy *via* iR-30a-mediated suppression of the autophagy regulators beclin-1, Atg12, and LC3II/LC3I (99). Blocking miRNA-30 upregulation can result in increased cardiomyocyte apoptosis due to controlling autophagy (92, 101). miRNA-30-5p maintains calcium/calmodulin-dependent PKA signaling and is lowered during cardiac hypertrophy (69, 95). On a contrasting note, increased levels of circulating miR-144 can increase autophagy by suppressing the autophagal negative regulator pmTOR, but which results in the same impacts of decreasing infarct area and increasing cardiac function (92). miR-144 can offer a degree of cardioprotection (36, 99). Increasing miR-144 leads to increased phospho-Akt, P-p44/42 MAPK, phospho-Glycogen Synthase Kinase 3 Beta, and autophagy signaling. Thus, there is reduced infarct size and better recovery, *via* the RIPC mechanism and miR-144 (99). Cardiac progenitor-derived miR-451 also offers a protective preconditioning effect on the heart that leads to better clinical outcomes (healing, protection, and post-MI neovessel formation) after acute ischemia, when miR-451 enriched exosomes were applied to ischemic rodent hearts (96, 101). They increased the formation of cardiac fibroblasts that promoted anti-fibrosis, increasing angiogenesis, and cardiomyocyte survival (101). Mesenchymal stem cell (MSC) derived-exosome reduced infarct size by 45% and reduced inflammation in a murine IRI model (91–93, 100, 109). MSC-exosomal miRNA also increased the density of working capillaries after MI in a rat model, allowing for more blood flow. Promotion of endogenous angiogenesis was *via* activation of the nuclear factor-κB pathway and transferring STAT3 (transcriptional activator) into the cell (102). GATA-4 MSC-derived exosomes transplanted onto the rat heart also decreased infarct size and increased contractility of the heart (93). PDGF-D (platelet-derived growth factor-D) is also an important factor *via* which MSC-exosomal miRNAs initiate their positive impacts. Akt-overexpressing MSC-derived exosomes increase EC migration, proliferation, *in-vitro* tube formation, and *in-vivo* neovascularization (102). miRNAs taken from ischemic cardiac tissues can be given to the infarcted myocardium through exosomes, to control autophagy, as a protective factor against apoptosis (101).

Ultimately, in AMI, exosomes play a protective role *via* reprogramming and initiating exosome-mediated crosstalk between cardiac and BM cells (99), changing the cell phenotype and BM microenvironment and impacting distant environment biology, leading to permanent re-education. There is also an adaptive role of increased cardiac cell-derived exosomal miRNAs in the blood, as it warns other cells of heart damage. Of all the cardiac-specific miRNAs released by cardiomyocytes, miR-1, 133a/b, 208a, and 499-5p are the ones that are elevated the most during MI, while miRNA-122 and 375 levels are lowered (92, 96). These upregulated miRNAs maintain cardiogenesis and heart function. In AMI, the presence of these miRNAs in the plasma indicates heart dysfunction. For example, the concentration of miR-208b in the plasma can be increased 1600x or 3000x after AMI, compared to healthy controls. miR-208 and miR-499 control sarcomeric genes, whereas miR-1 and miR-133a control ion channel genes that regulate the conductance, contractile rhythm, and automaticity of the heart (96). Since cardiac exosomal miRNAs are stable, protected from RNase degradation in the plasma, can be present in urine (unlike troponin T), with miRNA levels were elevated in MI, independent of myocardial apoptosis and sometimes released even before troponin T (96, 97) (<4 h after MI), they are reliable biomarkers of AMI. There are higher sensitivity and specificity to measure levels of cardiac-specific miRNAs compared to troponin T for cardiac pathology (96).

Exosome-mediated intercellular communication between cardiomyocytes and cardiac fibroblasts exhibited miR-208a-regulated cardiac fibrosis post-MI (MI). *In-vitro*, Hypoxia-stimulated (CM-Hypo) or Angiotensin II (Ang II)-treated cardiomyocytes (CM-Ang II) showed increased expression of miR-208a. Elevated expression of miR-208a was observed in cardiac tissues of murine models of MI and DOX-induced cardiomyopathy. Transfer of miR-208a from CM-Hypo or CM-Ang II to cardiac fibroblasts *via* exosomes promoted cardiac fibroblast proliferation and myofibroblast differentiation. Intramyocardial administration of exosomes derived from CM-Hypo or CM-Ang II led to the elevated cardiac miR-208a expression, decreased cardiac function, increased cardiac fibrosis in rats. Inhibition of miR-208a in post-MI rats attenuated cardiac fibrosis, improved cardiac function suggesting the role of cardiomyocyte-derived exosomal miR-208a in the progression of fibrosis post-MI. The pro-fibrotic effects of miR-208a are mediated by targeting Dyrk2, which *via* phosphorylation of nuclear factor of activated T-cells (NFAT) prevents its nuclear transfer for induction of fibrosis (116). Similarly, another study investigating the effect of cardiac macrophage-derived exosomes enriched with miR-155 on fibroblasts showed suppression of fibroblast proliferation and increased fibroblast inflammation. *In-vivo*, elevated expression of miR-155 was observed in cardiac macrophage post-MI. Administration of exosomal miR-155-derived from macrophage led to cardiac rupture and deficiency of miR-155 attenuated improved cardiac function with decreased incidence of cardiac rupture after acute MI (117).

The exosome-mediated intracellular signaling between cardiac progenitor cells (CPCs) has been increasingly investigated as a crucial cardiac regenerative mechanism post-ischemic injury. CPCs are a heterogeneous population of cells capable of differentiating into different cell types such as cardiomyocytes, SMCs, and ECs. Various studies reported improvement of LV function and reduction in scar size after intra-myocardial transplantation of CPCs after MI injury in preclinical models (118–121). An *in-vitro* study demonstrated CPC-derived exosomes in the enhancement of endothelial cell migration *via* an extracellular matrix metalloproteinase inducer (EMMPRIN)-mediated mechanism. The exosomes released by CPCs upon transplantation resulted in increased capillary density and promoted angiogenesis (122). Another study reported CPCs derived exosomes in cardioprotection from acute ischemia/reperfusion (MI/R) injury. While *in-vitro* treatment of cardiomyoblasts (H9C2) cells with CPCs-exosomes protected from oxidative stress by inhibition of caspase 3/7 activation, *in-vivo* delivery of CPCs-exosomes in myocardial ischemia/reperfusion murine model resulted in inhibition of cardiomyocyte apoptosis (123).

Circulating RNAs (circRNAs) are a class of endogenous non-coding RNAs that regulate gene expression in eukaryotes. Exosomal circHIPK3 derived from hypoxia-pretreated cardiomyocytes regulates oxidative damage in cardiac microvascular endothelial cells (CMVECs) *via* the miR29a-mediated IGF-1 expression. This suggested exosomal circHIPK3 as a potential target in cardiomyocytes to control CMVECs dysfunction under oxidative conditions (124). Dou et al. demonstrated mechanistic regulation of endothelial cell function by vascular smooth muscle cells in ischemia. Vascular smooth muscle SIRT1 inhibited endothelial angiogenic activity induced by hypoxia *via* the exosomal circRNA cZFP609. Upon delivery to ECs, cZFP609 detains HIF1α in the cytoplasm and thereby inhibits VEGFA expression and endothelial angiogenic functions (125).

Circulating exosomes from patients with ST-segment-elevation MI were enriched with sphingolipid species such as ceramides, dihydroceramides, and sphingomyelins. Significantly increased Ceramide, dihydroceramide, and sphingomyelin exosome content correlated with cardiac troponin, leucocyte count, and lower left ventricular ejection fraction (126).

In an *in-vitro* model of hypoxic cardiomyocytes, Wang & Zhang showed the effect of exosomal lncRNA AK139128 on cardiac fibroblasts (CFs) proliferation, migration, and invasion. Exosomal lncRNA AK139128 derived from hypoxic cardiomyocytes stimulated CF apoptosis *in-vitro* and exacerbated MI in a rat model by promotion of CFs apoptosis and inhibition of cell proliferation (127). Circulating levels of exosomal lncRNAs had been assessed in patients with acute MI as potential biomarkers (128, 129). Chen et al. reported higher serum expression of lncRNA NEAT1 and MMP9 in patients with acute MI compared to patients with unstable angina and non-MI. Another study evaluating circulating lncRNAs as prognostic biomarkers reported significantly upregulated exosomal lncRNAs ENST00000556899.1 and ENST00000575985.1 in patients with acute MI. The association between upregulated ENST00000575985.1 with clinical parameters such as inflammatory biomarkers, prognostic indicators, and myocardial damage markers suggested a positive association of exosomal lncRNAs ENST00000575985.1 with the risk of HF (129).

### Cardiomyopathy

Cardiomyopathies represent a group of diseases of myocardium-associated dysfunction, which can lead to cardiac morbidity and mortality due to progressive HF. Depending on structural and functional changes, cardiomyopathies are classified as dilated cardiomyopathy (DCM), hypertrophic cardiomyopathy (HCM), restrictive cardiomyopathy (RCM), and arrhythmogenic cardiomyopathy (130, 131). These different groups of cardiomyopathies are further classified based on pathogenesis, such as systemic inflammation, infection, or inherited disease-associated cardiomyopathies. Exosomes have been implicated in cardiomyopathies due to their pro-inflammatory attributes during the onset of different cardiomyopathies.

#### Dilated Cardiomyopathy

**Figure 2:**
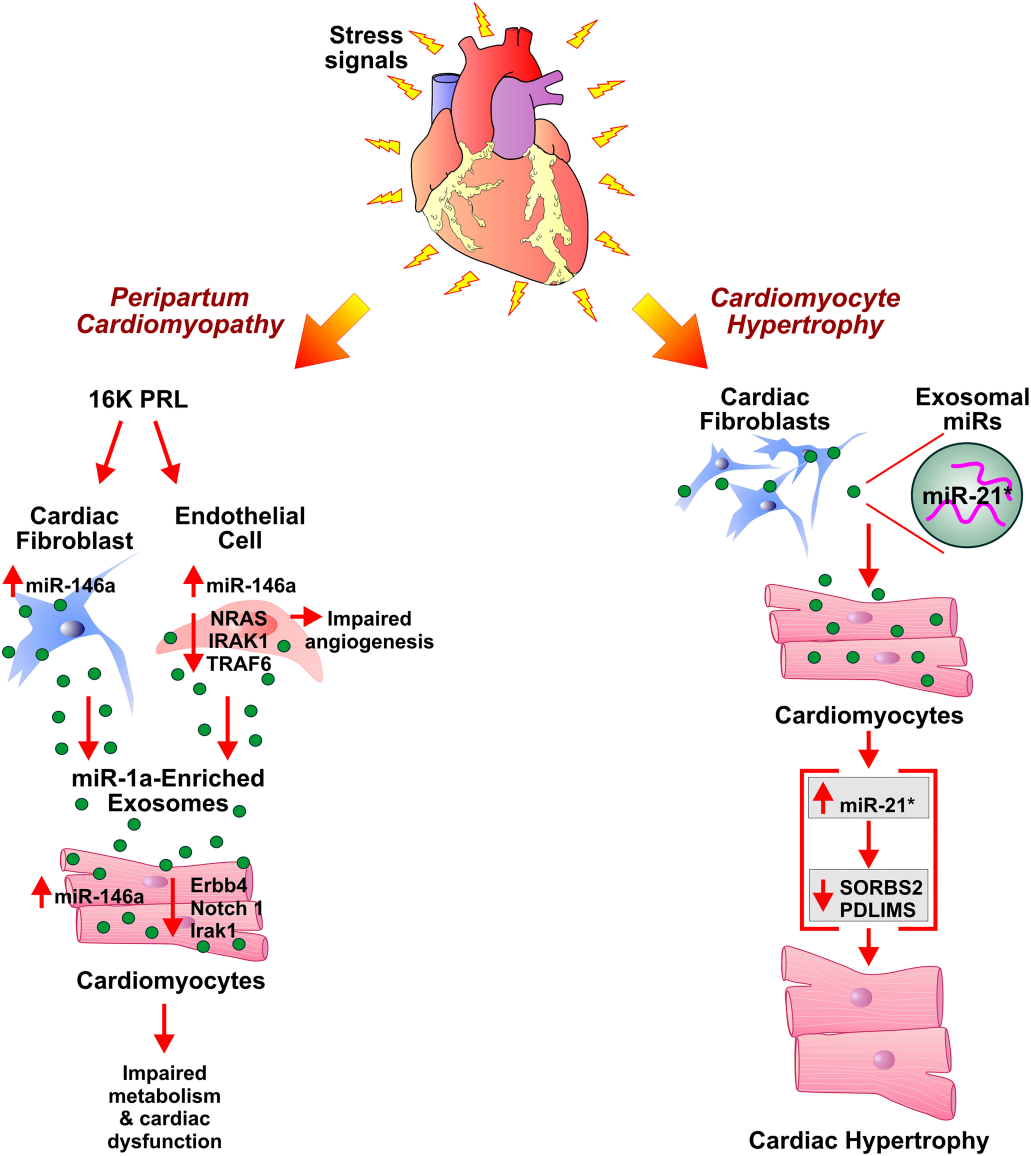
Role of exosomes in development of peripartum cardiomyopathy and cardiac hypertrophy. Antiangiogenic 16 kDa fragment, 16K PRL, is generated in peripartum cardiomyopathy patients by cathepsin D-mediated cleavage of prolactin (PRL). 16K PRL stimulates cardiac fibroblasts and endothelial cells to secrete miR-146a enriched exosomes which upon transferring miR-146a to cardiomyocytes lead to impaired metabolism and angiogenesis in cardiomyocytes. miR-21*-enriched exosomes secreted by cardiac fibroblasts, under stress conditions, elevate miR-21* in cardiomyocytes. This results in cardiomyocyte hypertrophy by downregulation of SORBS2 and PDLIM5 expression levels.

One of the most common types of cardiomyopathies, dilated cardiomyopathy (DCM), is characterized by an increase in myocardial mass and volume. It is generally associated with the enlarged left ventricle and contractile dysfunction. Thirty five percentage of DCM cases are attributed to genetic causes, and acquired causes include hypertension, infection, inflammation, and endocrine disturbances. The limited literature on the role of exosomes in the pathogenesis of DCM warrants further investigation of the interplay between various cell-derived exosomes and its effect on cardiomyocyte and thus left ventricular remodeling characteristics of DCM. Serum-derived exosomes from pediatric DCM upregulated the expression of ANP and BNP in primary cardiomyocytes. The inhibition of exosome uptake by cytochalasin D ameliorated alteration in gene expression, further corroborated exosome-mediated cardiomyocyte hypertrophy. Though the study did not investigate the *in-vivo* effect of DCM serum-derived exosome or exosomal cargo contributing to gene expression alteration, it explained the exosome-mediated, renin-angiotensin-aldosterone system (RAAS) and adrenergic system independent, pathological hypertrophy in cardiomyocytes (132). Other studies investigated cardiac miRNA-mediated regulation leading to DCM. Circulating levels of fibrosis-linked microRNAs: miR-21, miR-26, miR-29, miR-30, and miR-133a have been implicated in DCM. These miRNAs, specifically miR-133a, have been correlated with collagen synthesis markers, fibrosis controlling factors, and MMPs/TIMPs system involved in extracellular matrix metabolism. This provided necessary cues for future studies exploring the association between miRNA and fibrosis in DCM (133). Bang et al. demonstrated fibroblast-derived miR-21*-enriched exosomes in the development of DCM and cardiac hypertrophy (Figure 2). Loss of PDZ and LIM domain 5 (PDLIM5) has been implicated in DCM pathogenesis as PDLIM5 plays a pivotal role in the structure and function of striated muscle (134). Cardiac fibroblast-derived exosome enriched with miR-21* regulates expression of PDLIM5 in cardiomyocytes. Silencing of PDLIM5 by fibroblast-derived miR-21* suggested intercellular communication between cardiac fibroblasts and cardiomyocytes affecting cardiac muscle structure and function (77). Angiotensin II (Ang II) stimulated cardiac fibroblasts (CFs) lead to pathological remodeling in cardiomyocytes *via* a paracrine mechanism. *In-vitro* Ang II treatment of CFs enhanced exosome secretion by activation of Ang II receptor types 1 (AT1R) and 2 (AT2R). CFs derived exosomes enhanced Ang II production by cardiomyocytes by upregulation of renin, angiotensinogen, AT1R, and AT2R and downregulation of angiotensin-converting enzyme 2 expressions, thus resulting in cardiomyocyte remodeling. Proteomic analysis revealed the association of exosomal proteins with PI3K/Akt and MAPK pathways, hypertrophic cardiomyopathy, and dilated cardiomyopathy. The CF-derived exosomes activated mitogen-activated protein kinases (MAPKs) and Akt and thus upregulated RAS in cardiomyocytes. Treatment with GW4869 and DMA resulted in a reduction in Ang II-induced exosome secretion from CFs and pathological remodeling in cardiomyocytes. Thus, the study demonstrated Ang II-mediated stimulation of CFs exosome secretion and CFs exosome-mediated cardiomyocytes remodeling leading to hypertrophy and HF (79). Other studies elucidated alteration in endogenous miRNAs expressions such as miR-133a, miR-1, miR-25 miR-30c in cardiomyocytes, and its association with the development of dilated cardiomyopathy (135–138). Exosome-mediated crosstalk between various cells and cardiomyocytes in the development of dilated cardiomyopathy still needs scientific attention.

#### Diabetic Cardiomyopathy

**Figure 3:**
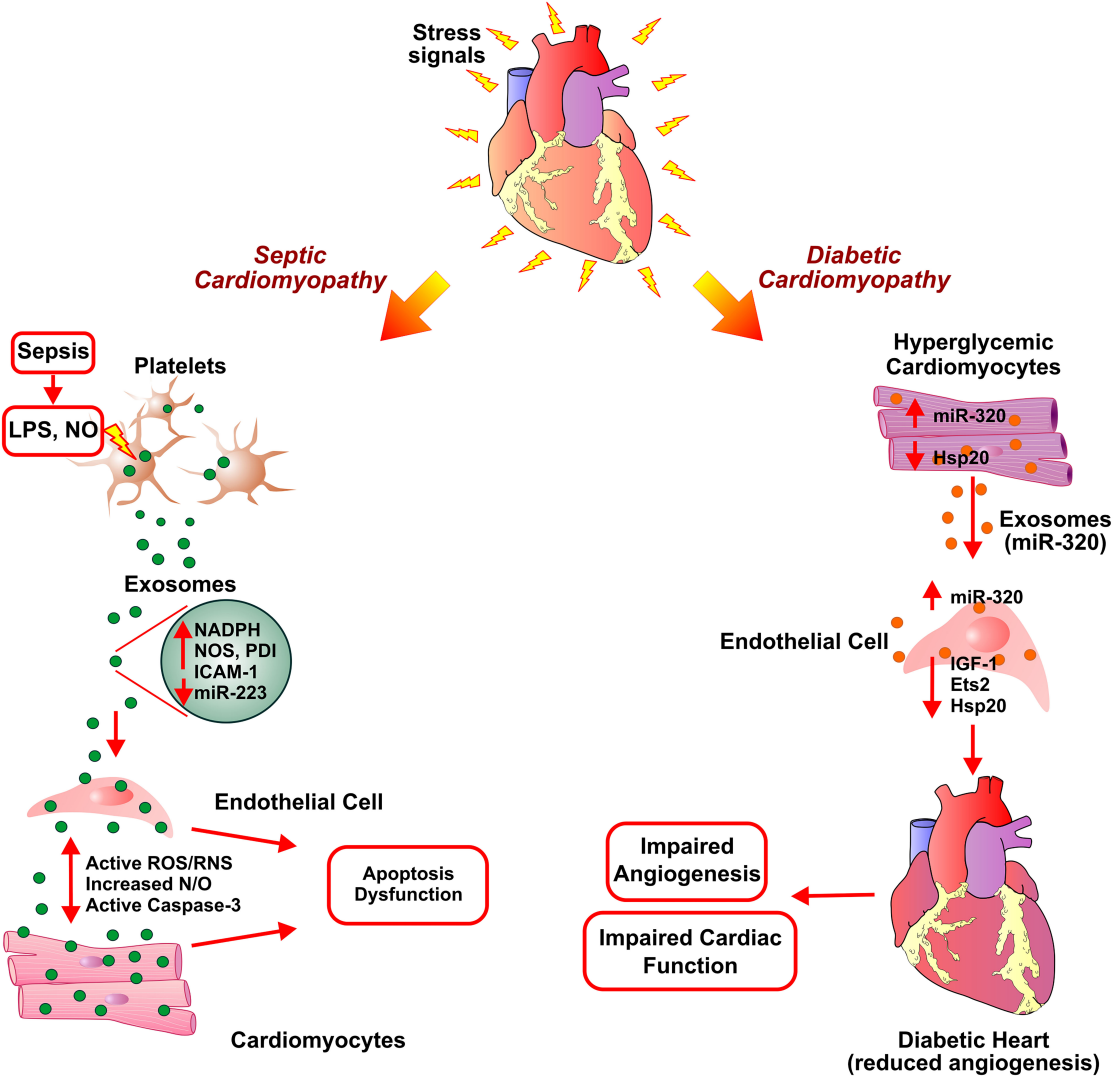
Exosome-assisted development of septic cardiomyopathy and diabetic hypertrophy. In response to high levels of lipopolysaccharide (LPS) and nitric oxide (NO) in sepsis, platelets secrete inflammatory exosomes with reduced miR-233 and are enriched in NADPH oxidase, nitric oxide synthases (NOS), and protein disulfide isomerase (PDI). This induces apoptosis in endothelial cells and cardiomyocytes *via* activation of reactive oxygen/ nitrogen species (ROS/RNS) signaling pathways, caspase 3 activation, thereby leading to cardiomyopathy. Exosomes secreted from diabetic cardiomyocytes contain increased levels of miR-320. Uptake of exosomal miR-320 by endothelial cells results in increased levels of Hsp20, IGF-1, and Ets2, thus, inhibiting angiogenesis in diabetic hearts.

Diabetes mellitus is a metabolic disease clinically identified as high blood glucose levels either due to lack of (Type I) or resistance to insulin (139). In the mammalian heart, ECs play an important role in cardiomyocyte survival and myocardial contraction (140). The high blood glucose levels in diabetes can result in endothelial dysfunction and microvascular rarefaction (141, 142). Diabetic Cardiomyopathy (DbCM) is associated with structural and functional alterations, such as insufficient myocardial angiogenesis, as a complication of diabetes (143). As an exact regulatory mechanism of ECs functions and its crosstalk with cardiomyocytes in response to hyperglycemic conditions is under investigation, several recent studies suggested cardiomyocytes derived exosome cargo, miRNAs, mRNAs, and proteins, play a key role in modulating functions of coronary ECs (144–146). While significant inhibition of proliferation and migration was observed when ECs were co-cultured with cardiomyocytes derived from type 2 diabetic Goto-Kakizaki (GK) rats compared to co-culture without cardiomyocytes from non-diabetic Wistar rats (147). This observation pointed toward the beneficial effects of cardiomyocyte-mediated regulation of endothelial function in normal conditions and the role of cardiomyocyte-derived factors, including exosomes, in diabetic endothelial dysfunction. The anti-angiogenic effects of GK-cardiomyocyte-derived exosomes were abolished by the treatment of co-culture with an inhibitor of exosome formation/release, GW4869. Though exosomes derived from both GK-cardiomyocytes and WT-cardiomyocytes showed molecular markers (CD63 and CD81), they exhibited contrary regulatory function in the angiogenesis of ECs. Further, the diabetic GK cardiomyocytes derived exosomes showed high levels of miR-320, and lower levels of miR-126 and Hsp20 proteins compared to exosomes derived from WT- cardiomyocytes. The transfer and uptake of miR-320 enriched exosomes from myocytes to ECs showed downregulation of miR-320 targets such as IGF-1, Hsp20, and Ets2 (Figure 3). Overexpression of miR-320 inhibited the proliferation and migration of recipient ECs. The study provided mechanistic insight into the impairment of myocardial angiogenesis in diabetes due to anti-angiogenic effects of exosomes derived from cardiomyocytes (147). The mechanism of diabetes-mediated activation of miR-320 is still obscured and may not be dependent on hyperglycemia as high glucose levels reported to inhibit miR-320 expression in HUVECs (148). Contrarily, exercise exosome enriched with miR-29b and miR-455 lead to downregulation of MMP9 and protect from the detrimental effects of MMP9 such as matrix degradation, fibrosis, and myocyte uncoupling in diabetes (149).

Communication between cardiomyocytes and ECs is an integral part of the maintenance of metabolic requirements of the heart, as recent research showed cardiomyocyte-derived exosomes in the regulation of glucose uptake by ECs. Cardiomyocytes under glucose-deprived conditions secreted exosomes enriched in glucose transporters and enzymes involved in glycolysis. Uptake of these exosomes by ECs leads to an increase in glucose uptake, glycolysis, and the production of pyruvate and thus suggested exosome-mediated cell communication being involved in metabolic regulation (150). Recently, Wang et al. explained the role of cardiomyocyte-derived exosomes and their cargo in cardioprotection in diabetes. The study reported heat shock protein (Hsp) 20-dependent stimulation of exosomes production by interaction with an upstream protein involved in exosome biogenesis i.e., Tsg101. Administration of neutral sphingomyelinase/ceramide inhibitor, GW4869 in *in-vivo* diabetes mouse model, resulted in impaired Hsp20 mediated improvement in cardiac function. The study suggested Hsp20 transgenic cardiomyocytes-derived exosomes and their paracrine effect in Hsp20 mediated cardioprotection against diabetes-induced cardiac injury (151). Using clinical samples and a murine model of obesity, the study elucidated the physiological role of liver-secreted miR-122 in cardiac metabolic disorder. Circulating levels of exosomal miR-122 from clinical samples as well as obese mice were positively correlated with impaired cardiac function indicated by reduced ejection fraction (EF) and increased NT-proBNP. Treatment of murine cardiomyocytes with human plasma exosomes impaired mitochondrial ATP production and oxygen consumption *via* the transfer of miR-122. Similarly, altered cardiac structure and function with impaired mitochondrial function were observed due to an increase in hepatic and circulating exosomal miR-122 in diet-induced obese mice. Deleterious effects on cardiac structure and function were attenuated by liver-specific blockage of miR-122. By binding to the 3′-UTR region and suppression of ADP-ribosylation factor-like 2 (Arl-2), a regulator of mitochondrial ATP production, miR-122 affected cardiac energy homeostasis and thus led to the development of metabolic cardiomyopathy (152). The hippo pathway participates in the regulation of apoptosis, autophagy, and organ size. Mammalian sterile 20- like kinase 1 (Mst1), a component of the Hippo pathway has been reported to be involved in the development of DbCM. *In-vitro* and *vivo* experimentation suggested Mst1 transfer *via* exosomes from cardiac microvascular endothelial cells (CMECs) to cardiomyocyte (CM) contributed to pathogenesis to DbCM. The diabetic model of endothelial-specific Mst1 transgenic mice showed deteriorated cardiac function and aggravated insulin resistance. Uptake of CMECs-derived exosomal Mst1 by CMs inhibited autophagy and enhanced apoptosis. Under diabetic conditions, increased binding of Mst1 and Daxx resulted in inhibited glucose uptake by the disruption in the glucose transporter type 4 (GLUT4) membrane translocation through decreasing the interaction between Daxx and GLUT4. The study suggested pathogenic effects of diabetic CMECs-derived exosomal Mst1 on CM *via* autophagy inhibition, apoptosis promotion, and suppression of glucose metabolism leading to diabetic cardiomyopathy (153).

The circRNA derived from human umbilical vein endothelial cell exosomes (HUVEC-Exos) under hyperglycemic conditions regulates the senescence of VSMCs. circRNA 0077930 from hyperglycemia-stimulated vascular endothelial cell exosomes induced VSMCs senescence by down-regulation of miR-622 expression and up-regulation of Kras, p21, p53, and p16 expression (154).

#### Peripartum Cardiomyopathy

Peripartum cardiomyopathy (PPCM) is pregnancy-associated cardiomyopathy characterized by left ventricular dysfunction leading to HF during the late peripartum period and/or few months of postpartum (155, 156). Cardiac Cathepsin D (CD) mediated proteolytic cleavage of the nursing hormone prolactin (PRL) into a 16-kDa N-terminal prolactin fragment (16K PRL) has been proposed as a pathogenic factor in the onset of PPCM (Figure 2). The aggravation of detrimental effects of oxidative stress and activated CD by PRL results in myocardial hypoxia and apoptosis, thus facilitate the initiation of PPCM development (157). Treatment of HUVECs with 16K PRL resulted in NF-κB mediated upregulation of miR-146a leading to inhibition of angiogenesis. 16K PRL also promoted the release of miR-146a enriched exosomes from ECs. The ECs derived miR-146a enriched exosomes can be taken up by cardiomyocytes which in turn resulted in the elevation of miR-146a levels. The expression of downstream factors of miR-146a such as Notch1, Erbb4, and Irak1 was decreased in cardiomyocytes which triggered cellular mitogenesis and differentiation. The study suggested exosome-mediated transfer of miRNA as an intercellular communication mechanism between ECs and cardiomyocytes in PPCM. ECs and non-myocyte cardiac cells, such as cardiac fibroblasts of cardiomyocyte-specific *Stat3* knockout mice heart showed an increased level of miR-146a. This suggested apart from ECs, fibroblasts as a potential source of 16K PRL induced secretion of miR-146a enriched exosomes in PPCM. Furthermore, significantly higher plasma levels of exosomal miR-146a were observed in patients with acute PPCM compared to healthy postpartum controls and patients with dilated cardiomyopathy. Blockade of PRL using the standard treatment for HF (beta-blocker and ACE inhibitor) and bromocriptine lead to the recovery of PPCM patients with normalization of circulating exosomal miR-146a levels. The study suggested the potential utility of miR-146a as a specific biomarker for PPCM (158).

#### Septic Cardiomyopathy

Sepsis is an infection-induced severe inflammatory response and a leading cause of intensive care unit (ICU) death. It is associated with multiple organ dysfunctions with sepsis-induced cardiomyopathy being the leading contributor to septic death (159, 160). The reactive immune response against an infection triggers sepsis and subsequently affects multiple vital organs such as the heart, kidney, etc. (161). Blood clotting due to the sepsis-induced inflammation results in obstruction of blood and nutrient supply to the heart hence leading to cardiovascular dysfunction. Cardiovascular complications, such as decreased ejection fraction and biventricular dilation, had been reported in septic patients (162). Circulating proinflammatory cytokines (IL-6 and TNFα) and are reactive oxygen/nitrogen species (superoxide, nitric oxide, and peroxynitrite) implicated in inducing cardiomyopathy in sepsis (161). Though the exact mechanism of onset and progression of septic cardiomyopathy is still unknown, studies reported infected macrophage-derived exosomes in mounting inflammatory response and as a cause of cardiovascular dysfunction in sepsis (163–165). Treatment of RAW 264.7 macrophage cell line with LPS leads to an increase in the secretion of pro-inflammatory exosomes, which resulted in elevated levels of IL-1β and IL-6. Inhibition of exosome generation by treatment with GW4869, a neutral sphingomyelinase inhibitor, diminished pro-inflammatory cytokine production in macrophages and reduced LPS-stimulated pro-inflammatory cytokine production and cardiac inflammation in mice. The study corroborated the functional association of exosomes in sepsis-induced myocardial dysfunction by blockade of exosome generation using GW4869 (166). In an *in-vitro* study investigating mechanistic involvement of platelet-derived exosomes in endothelial cell apoptosis and development of septic vascular dysfunction, authors described the redox reactive role of platelet-derived exosomes in vascular dysfunction in sepsis. The study reported that the release of platelet-derived exosomes in sepsis was triggered by increased nitric oxide (NO) generation and exposure to lipopolysaccharide (LPS). The platelet-derived septic exosomes showed high content of NADPH oxidase, nitric oxide synthases (NOS), and NADPH oxidase regulatory protein disulfide isomerase (PDI). Further, these septic-like platelet-derived exosomes and NO promoted generation of active ROS/RNS by NADPH oxidase and NO synthase type II leading to caspase-3 activation and subsequently apoptosis of target ECs (Figure 3). The study thus suggested the pro-apoptotic effects of exosomes in ECs from sepsis patients (164). In another study, the treatment of isolated rabbit heart and rat papillary muscle preparations with platelet-derived exosomes from septic patients resulted in reduced myocardial contractility and pre-exposure to LPS exacerbated the detrimental effects of exosomes. Further, Nitric oxide (NO) generation by exosomes induced myocardial NO generation suggesting an exosomal NO-mediated novel mechanism of myocardial dysfunction in sepsis (163). The downregulation of expression of Sema3A and Stat3 by Mesenchymal stem cells (MSCs) derived miR-223 enriched exosomes have been implicated in cardioprotection in sepsis as exosome-mediated downregulation leads to the reduction in macrophage inflammation and cardiomyocyte cell death. This suggested miR-223 dependent protective mechanism in sepsis *via* MSC-derived exosomes (167). Some of the studies reported reduced serum levels of miR-223 in sepsis patients compared to healthy controls or in non-surviving sepsis patients compared to surviving sepsis patients (168, 169). miR-223 had been implicated in the regulation of pro-inflammatory markers such as IL-6, IL-1β, and ICAM-1 (170–172). Thus, reduced expression of miR-223 in sepsis may lead to increased expression of these pro-inflammatory proteins and promoting myocardial depression *via* enriched exosome. Thus, septic cardiomyopathy may be attributed due to the accumulation of sepsis-associated exosomes and cargo contents of exosomes such as miRNAs, NOS, etc.

#### Uremic Cardiomyopathy

Described as a complication of chronic kidney disease (CKD), uremic cardiomyopathy (UCM) is characterized by hypertension, cardiac hypertrophy, inflammation, and fibrosis. It accounts for approximately 50% of deaths due to CKD (173–175). Under uremic conditions, macrophage-derived exosomes *via* the transfer of miR-155 promoted pyroptosis in the heart. Exosomal miR-155 targeted and suppressed transcription of Forkhead Transcription Factor 3a (FoxO3a). Overexpression of FoxO3a or inhibition of miR-155 in uremic mice ameliorated pyroptosis and improved UCM, as evidenced by reduced heart weight, size, myocardial hypertrophy, interstitial fibrosis area with improved cardiac function. *In-vitro*, blockage of macrophage-derived exosomal miR-155 using GW4869 attenuated pyroptosis and improved UCM in cardiomyocytes indicated by reduced pro-inflammatory markers and apoptosis. This was the first report addressing the pathological contribution of pyroptosis mediated by macrophage-derived exosomal miR-155 in the development of UCM (176).

### Pulmonary Arterial Hypertension (PAH)

Pulmonary arterial hypertension (PAH) is a rare disease characterized by progressive vascular remodeling of the pulmonary artery and increased pulmonary vascular resistance due to vasoconstriction and subsequently right ventricular (RV) failure (Figure 1). The remodeling in PAH includes apoptosis and proliferation of pulmonary vascular ECs, activation of SMCs and fibroblasts, perivascular inflammation, and extracellular matrix protein deposition (177, 178). PAH is clinically classified as idiopathic (IPAH), heritable (HPAH), and PAH associated with other diseases (APAH). Exosome-mediated miRNA crosstalk between pulmonary artery smooth muscle cells (PASMCs) and pulmonary arterial endothelial cells (PAECs) have been implicated in migratory phenotypes of SMCs and ECs and hence in the pathogenesis of PAH. PASMCs exposed to PAH stimuli showed increased levels of miRNA-143-3p, leading to an increase in cell migration of PASMCs. PASMCs derived exosome-mediated transfer of miRNA-143-3p also promoted migration and proliferation of PAECs. Upregulation of miRNA-143-3p expression was observed in the lung and right ventricle of mice exposed to hypoxia and in primary PASMC from PAH patients. The expression of miR-143-3p in cardiomyocytes and cardiac fibroblast cells suggested interaction of PASMCs derived exosome enriched in miR-143-3p with cardiomyocytes and fibroblasts in the heart leading to progression of PAH-induced right-sided HF. Further, genetic deletion and pharmacological inhibition of miR-143 in the mice model prevented the development of PAH and mitigating the effect of hypoxia-induced PH after induction of hypoxia. The study thus suggested paracrine signaling mediated by miR-143-3p as a vital modulator of pulmonary vascular remodeling in the pathogenesis of PAH (179).

Increased levels of EVs and their association with pulmonary vascular resistance and vascular dysfunction had been reported in patients with PAH. To explain the mechanistic role of EVs in PAH, Aliotta et al. (180) studied the pathogenic effects of EVs from monocrotaline (MCT)-treated mice in PAH development. Authors reported lung-derived EVs (LEV), and plasma-derived EVs (PEV) from MCT-treated mice lead to the development of MCT-induced pulmonary hypertension in healthy mice evidenced by increased right ventricular mass and pulmonary vascular wall thickness. EVs exerted pathogenic effects either by directly contributing to pulmonary vascular remodeling or by inducing bone marrow cell differentiation into endothelial progenitor cells which affected pulmonary vasculature (180). Further Aliotta et al. (181) showed that exosome fraction, but not microvesicle fraction of EVs derived from mice with monocrotaline (MCT)-induced pulmonary hypertension (PH) induced PH in healthy mice (181). Increased levels of miRs-19b,-20a,-20b, and−145 have been observed in exosomes derived from MCT induced PH murine model and PAH patients of which miR-145 has been already reported to be involved in the pathogenesis of PAH in both animal model and PAH patients (182). The exosomal fraction of mesenchymal stem cells (MSCs) derived EVs modulated protective effects against MCT-induced PH by anti-inflammatory and anti-proliferative miRs such as miRs-34a,−122,−124, and−127. The study suggested the function of exosomes in both the development and reversal of pulmonary vascular remodeling and right ventricular hypertrophy in the murine PH model depending on cell origin and miRNA cargo (181). Mesenchymal stromal cell (MSC)-derived exosomes (MEX) exerted a protective effect on PASMCs and PAECs by inhibition of pro-proliferative signal. In the hypoxia-induced PH murine model, the administration of MEX inhibited activation of hypoxic signaling by suppression of the hypoxic activation of signal transducer and activator of transcription 3 (STAT3) phosphorylation underlying pulmonary inflammation. This resulted in increased expression of miR-204 in the lungs, which is reported to be downregulated in a murine model of PH and human pulmonary hypertension (183). MEX downregulated the expression of miR-17 which is regulated by STAT3 suggesting that MEX is a key modulator of hyperproliferative signaling by inhibition of STAT3 in hypoxia-induced pulmonary hypertension (184).

The pathological vascular remodeling associated with PAH has been attributed to an imbalance between cell proliferation and apoptosis. Translationally controlled tumor protein (TCTP) is a pro-survival and anti-apoptotic mediator that has been implicated in microtubule stabilization and secretion of proteins through exosomes (185, 186). Transfer of exosomal TCTP derived from blood outgrowth endothelial cells (BOECs) have functional consequences on pulmonary artery smooth muscle cells (PASMCs). The overexpression of TCTP in PASMCs induces hyperproliferation and reduced apoptosis as evident in PAH (187). Widely distributed in the pulmonary vasculature, the lipid-peroxidizing enzyme 15-lipoxygenase2 (15-LO2) metabolizes arachidonic acid to 15-hydroxyeicosatetraenoic acid (15-HETE). PAECs and pulmonary artery smooth muscle under hypoxia express 15-LO2 and its upregulation leads to cell proliferation, anti-apoptosis, and vasoconstriction, and thus PAH. Under hypoxic conditions, pulmonary artery endothelial cells (PAECs) showed increased secretion of exosomes enriched in 15-LO2 which subsequently activates the STAT3 signaling pathway, thus results in hyperproliferation of PAECs (188).

### Valvular Heart Disease (VHD)

Valvular heart disease (VHD) is a leading cause of cardiovascular mortality and is associated with anatomical or functional abnormalities of cardiac valves. If untreated, VHD leads to progressive cardiac dysfunction. Stenosis and regurgitation/insufficiency represent major structural and functional aberration associated with VHD. Stenosis is characterized by the inadequate outflow of blood due to the narrowing of the Valvular orifice or failure of the valve to open completely. The inability of the valve in preventing backflow of blood due to impaired valve closure is termed regurgitation/insufficiency. VHD can be caused by congenital disorders or by acquired pathological conditions (189).

Exosomal miRNAs have been implicated in the regulation of the formation of the cardiac valve during development. Aberrant miRNAs mediated up/down-regulation of target genes involved in valve formation may lead to the pathogenesis of VHD. During the cardiac valve formation, endocardial cells undergo endothelial to mesenchymal transition and constitute a major part of cardiac jelly in the atrioventricular canal (190). Regulated by hyaluronic acid synthase2 (Has2), hyaluronan is an important extracellular matrix component of the endocardial cushion which is required for the formation and maintenance of the endocardial cushion. miR-23 acts as a negative feedback regulator of Has2 and prevents the excess deposition of hyaluronan in cardiac jelly. Aberrant miR-23 mediated regulation of Has2 leads to inhibition of the formation of hyaluronan and subsequently, cardiac valve defects in zebrafish (191). Using the mathematical model, authors showed endocardial cushion-derived exosomes for transfer of miR-23 for inhibition of endothelial-mesenchymal transition outside the atrioventricular canal region (190). Recently, Yang et al. reported an association between exosomal miRNAs and myxomatous mitral valve disease in the canine model. The expression levels of exosomal miRNAs involved in the regulation of cardiomyocyte energetics, fibrosis, and mitochondrial function such as miR-9, miR-181c, miR-495, and miR-599 were associated with valve disorder progression and congestive HF (192).

In response to pathogenic conditions, the phenotypic changes in Valvular interstitial cells (VICs) lead to calcified aortic valve disease due to extracellular matrix remodeling and mineral deposition. VICs can differentiate into either activated myofibroblast-like VICs or osteoblast-like VICs in hyperphosphatemic and pro-inflammatory conditions. The intercellular communication between VICs and Valvular endothelial cells (VECs) plays a vital role in valve homeostasis (193, 194). The uptake of VICs derived EVs by VECs influence its phenotype and subsequently into calcified valve phenotype (195). An *in-vitro* osteogenic environment containing elevated levels of calcium and phosphate leads to an increase in the expression of osteogenic markers such as Msx2, Runx2, PiT-1, TNAP, and Alpl. Similar to MVs derived from calcifying chondrocytes and VSMCs, the pro-calcified VICs derived EVs showed up-regulation of Annexins I, II, III, IV, V, VI, VII, and XI. The *in-vitro* study demonstrated EVs secretion with elevated calcium and annexin VI from rat VICs cultured with high calcium and phosphate and suggested a role in calcified aortic valve disease evidenced by co-localization of annexin VI with EVs in the aortic valve (196). Carrion et al. reported the mechanistic association between lncRNA HOTAIR and calcification of VSMCs in aortic valve disease. Cyclic stretch and WNT/ β-CATENIN modulate HOTAIR activity and repression of HOTAIR elevates expression of calcification-related genes, such as Bone Morphogenetic Protein 2 (BMP2) and alkaline phosphatase (ALP). Consequently, lncRNA HOTAIR plays a vital role in the calcification of VSMCs (197).

## Conclusion

Despite enormous research on paracrine effects of exosomes in intercellular communication, understanding of the function of secreted exosomes and exosome-mediated interaction among various cardiac cells in the pathological condition is still in the nascent stage. The cardiovascular function depends on orchestrated communication between cardiomyocytes and other cardiac cell types. Molecular cargo transferred by exosomes modulates cardiac homeostasis in physiological conditions. In pathological conditions, alterations in proteins, nucleic acids, and other cargo molecules transported by exosomes lead to the onset and progression of CVDs. Considering the major role of exosome-mediated paracrine signaling in the modulation of cardiac function in physiological and pathological conditions, further investigations should focus on sources of exosomes, their effect on neighboring cells, and alteration in cargo composition in cardiac pathology. The delineation of molecular mechanisms emphasizing these biological processes will help in the discovery of exosome-based diagnostic and potential therapeutic tools against cardiovascular pathologies.

## Author Contributions

AJ, AP, KG, and RS prepared the manuscript. AJ, AP, and VP wrote the main parts of the article and produced graphics. KG, RS, and VP reviewed and edited the manuscript. AJ and VP drafted the final version of the manuscript. All authors read and approved the final manuscript.

## Funding

This work received support from Canadian Institutes of Health Research (CIHR; Project Grant to VP; #PJT-165857), Natural Sciences and Engineering Research Council of Canada (NSERC; Discovery Grant to VP: #RGPIN/04766-2018), and Libin Cardiovascular Institute, Cumming School of Medicine (start-up operating funds to VP; postdoctoral scholarships to AJ, KG; and doctoral scholarship to RS).

## Conflict of Interest

The authors declare that the research was conducted in the absence of any commercial or financial relationships that could be construed as a potential conflict of interest.

## Publisher's Note

All claims expressed in this article are solely those of the authors and do not necessarily represent those of their affiliated organizations, or those of the publisher, the editors and the reviewers. Any product that may be evaluated in this article, or claim that may be made by its manufacturer, is not guaranteed or endorsed by the publisher.
